# Vestibular schwannoma associated with neurofibromatosis type 2: Clinical course following stereotactic radiosurgery

**DOI:** 10.3389/fonc.2022.996186

**Published:** 2022-09-16

**Authors:** Junhyung Kim, Yukyeng Byeon, Sang Woo Song, Young Hyun Cho, Chang-Ki Hong, Seok Ho Hong, Jeong Hoon Kim, Do Heui Lee, Ji Eun Park, Ho Sung Kim, Young-Hoon Kim

**Affiliations:** ^1^ Department of Neurological Surgery, Asan Medical Center, University of Ulsan College of Medicine, Seoul, South Korea; ^2^ Department of Radiology, Asan Medical Center, University of Ulsan College of Medicine, Seoul, South Korea

**Keywords:** neurofibromatosis type 2 (NF2), vestibular schwannoma (acoustic neuroma), stereotactic radiosurgery (SRS), tumor control, hearing preservation

## Abstract

**Objective:**

A lack of understanding of the clinical course of neurofibromatosis type 2 (NF2)-associated vestibular schwannoma (VS) often complicates the decision-making in terms of optimal timing and mode of treatment. We investigated the outcomes of stereotactic radiosurgery (SRS) in this population.

**Methods:**

We retrospectively analyzed NF2 patients treated with Gamma-Knife SRS for VS in our tertiary referral center. A total of 41 treated lesions from 33 patients were collected with a follow-up period of 69.1 (45.0-104.8) months. We reviewed the treatment history, hearing function, and other treatment-related morbidities in individual cases. We also analyzed pre- and post-treatment tumor volumes *via* imaging studies. Longitudinal volumetric analyses were conducted for the tumor volume response of the 41 treated lesions following SRS. The growth pattern of 22 unirradiated lesions during an observation period of 83.4 (61.1-120.4) months was separately evaluated.

**Results:**

Most treated lesions showed effective tumor control up to 85% at 60 months after SRS, whereas unirradiated lesions progressed with a relative volume increase of 14.0% (7.8-27.0) per year during the observation period. Twelve (29%) cases showed pseudoprogression with significant volume expansion in the early follow-up period, which practically reduced the rate of tumor control to 57% at 24 months. Among the patients with serviceable hearing, two (20%) cases lost the hearing function on the treated side during the early follow-up period within 24 months.

**Conclusions:**

Progressive NF2-associated VS can be adequately controlled by SRS but the short-term effects of this treatment are not highly advantageous in terms of preserving hearing function. SRS treatment candidates should therefore be carefully selected.

## Introduction

Neurofibromatosis type 2 (NF2) refers to a rare neoplastic syndrome of the nervous system, characterized by the presence of multiple schwannomas, meningiomas, or gliomas. NF2 results from the genomic aberrations in the *NF2* tumor suppressor gene, which regulates the production of merlin/schwannomin protein. More than half of the affected individuals are sporadic while some others are inherited in an autosomal dominant pattern. Currently, NF2 is clinically defined by the consensus diagnostic criteria without genetic testing. Most NF2 patients harbor vestibular schwannomas (VS), which often arise bilaterally and are more progressive at a younger age compared with sporadic cases.

For NF2-associated VS patients, the optimal timing and mode of treatment are still the subject of debate due to a biologic behavior that is distinct from their sporadic counterparts. Current clinical guidelines recommend diverse treatment options for individual cases, and stereotactic radiosurgery (SRS) is widely accepted as a viable therapeutic option (1, 2). However, a lack of understanding of the clinical course of NF2-associated VS often complicates the decision-making with regards to treatment. Some experts have insisted that a conservative observational approach (a wait-and-see strategy) is appropriate, especially in specific age groups (3, 4). In contrast, some others suggested early interventions with a treatment modality selected for each case (5). Several studies have reported that SRS is a valid and favorable treatment option that is as effective against VS in NF2 patients as in sporadic cases (6, 7).

We have here investigated the clinical outcomes of an SRS intervention for NF2-associated VS in a cohort from our institution. We evaluated the tumor growth patterns of individual cases and compared treated and untreated lesions.

## Materials and methods

### Study population

This study was a retrospective review of a case series of NF2 patients who underwent Gamma-Knife SRS treatment for VS between 1991 and 2021 at our tertiary referral center. All available clinical information and neuroimaging data were collected under the approval of our institutional review board. The study patients with NF2 had been identified *via* a clinical diagnosis that was based on the Manchester criteria (8). In cases with bilateral disease, each treated lesion was included as an individual case. Cases missing magnetic resonance image (MRI) data or a lack of detailed reports on the SRS treatment were excluded from the study. Cases lost to follow-up prior to 12 months from the date of the initial treatment were also excluded.

### Treatment modality and outcome assessment

The study patients were treated using the Leksell Gamma-Knife Perfection (Elekta, Stockholm, Sweden). In each case, 1 mm- or 1.5 mm-sectioned T1-weighted MRIs with gadolinium enhancement and 2 mm- or 3 mm-sectioned T2-weighted MRI scans were acquired for treatment planning. Doses were calculated using Leksell GammaPlan v5.34-11.3.1 (Elekta, Stockholm, Sweden) software. All patients were typically followed up initially at 3-, 6-, and 12-month intervals after the treatment, and then annually or biannually thereafter depending on their clinical course. Pretreatment screening and follow-up MRIs usually included 3 mm-sectioned axial and coronal postcontrast T1-weighted images, which were used for volumetric assessments of the tumor. The volumetric assessment was conducted by manual segmentation using 3D Slicer v5.10 (NA-MIC, Boston, MA), and the median value of the volume measurements from distinct sequences in each study session was used in the analyses.

For outcome assessments, tumor control, hearing preservation, and other treatment-related comorbidities were investigated. Tumor progression or recurrence was defined as a more than 10% increase in the tumor volume compared with pretreatment volume. Tumor pseudoprogression, or an early transient increase in the tumor volume after SRS, was not separately evaluated since it cannot be differentiated from true tumor progression in the first five years of follow-up (9, 10). Tumor control was defined as the arrest of tumor growth without progression or a decrease in the tumor volume. Hearing function was evaluated using pure tone audiometry and speech discrimination scoring for the patients who had serviceable hearing on the affected side prior to treatment. Hearing preservation was defined as retention of serviceable hearing (corresponding to a Gardner-Robertson grade I or II) at the last available audiometric follow-up.

### Statistics

Descriptive statistics were shown as a frequency with percentages for categorical data and as a median value with interquartile ranges (IQR) for continuous data. Differences between distinct phenotype groups were compared using the Chi-square test for categorical variables and the Wilcoxon-Mann-Whitney U-test for continuous variables. For the subset of patients with bilateral disease, tumor growth patterns were compared between the treated and untreated lesions. In generating tumor growth curves, the logarithmic relative tumor volumes were fitted by linear regression for individual cases, with an assumption of which would follow an exponential function as follows:


dV/dt=δ V

d(lnV)/dt=δ,

where *δ* is the effective growth rate.

All results were considered statistically significant if a two-tailed p-value was less than 0.05. All statistical analyses were performed using R v4.2.0 (R Foundation for Statistical Computing, Vienna, Austria).

## Results

### Baseline characteristics of the study subjects

**Figure 1:**
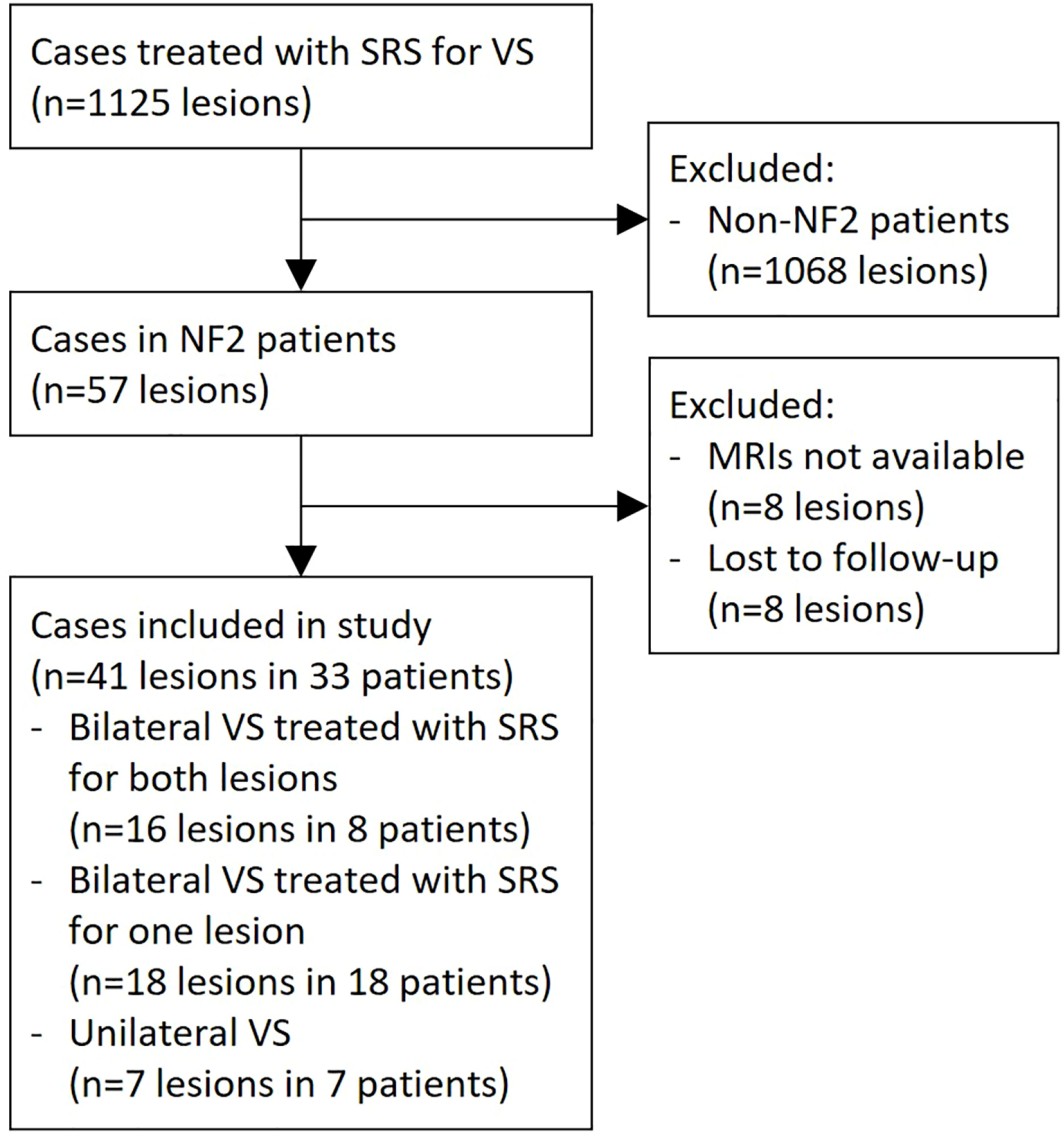
Selection of study subjects. NF2, neurofibromatosis type 2; MRI, magnetic resonance imaging; SRS, stereotactic radiosurgery.

A total of 33 NF2 patients were reviewed in this study (**Figure 1**). Bilateral VS was found in 26 (79%) patients in this case series, where 8 (24%) patients underwent SRS on both sides. Among the other cases with bilateral diseases, four (12%) underwent tumor resection without adjuvant SRS for the contralateral lesion, while 14 (42%) patients left one side untreated. Thereby, 41 lesions treated with SRS were included in the outcome assessment (**Table 1**).

**Table 1:** Baseline characteristics of the study patients.

	**VS (n=41) in NF2 patients**
Age at treatment (year)	37 [29, 50]
≥ 30	29 (71)
Sex	
Male	21 (51)
Criteria for NF2 diagnosis	
Bilateral VS	34 (83)
Unilateral VS with other multiple tumors	7 (17)
Phenotype	
Wishart	27 (66)
Feiling-Gardner	14 (34)
Laterality	
Left	17 (41)
Indication for treatment	
Primary	
Tumor growth	13 (32)
Tumor volume	21 (51)
Adjuvant or secondary (prior surgery)	7 (17)
Hearing function before SRS (Gardner-Robertson grade)	
I	2 (5)
II	8 (20)
III	3 (7)
IV or V	28 (68)

Values are numbers (%) or a median [range]. Data are given for each treated case. VS, vestibular schwannoma; NF2, neurofibromatosis 2; SRS, stereotactic radiosurgery.

While none of the current study patients were identified as inherited cases, only three of these patients received genetic counseling for NF2. One of these cases was shown to have a deletion of the *NF2* gene in a multiplex ligation-dependent probe amplification study. Based on a historical phenotypic categorization (6, 11, 12), 22 patients (27 lesions) were deemed to have the Wishart phenotype. The relationship between the presence of bilateral disease and specific phenotypes was not significant (p=0.292), whereas the age at the first treatment was significantly different between the two phenotypes: Wishart, 32 (23-42) years vs. Feiling-Gardner, 50 (43-56) years (p=0.002).

Most of the cases in our current series were treated with Gamma-Knife SRS as a primary intervention. Six cases received SRS as an adjuvant or secondary treatment after tumor resection. No patients were treated with bevacizumab during the follow-up period. More than half of our cases were treated due to a large tumor volume and had a non-serviceable hearing function at the time of their therapy. No other case of a cranial nerve impairment related to the target lesion was observed.

Gamma-Knife SRS was prescribed for a median tumor volume of 2.80 (0.70-5.90) cm^-3^ (**Table 2**). The majority of the treated lesions were Koos grade II (37%) or III (39%) with a maximal diameter of 2.0 (1.0-2.3) cm. Most cases in our case series underwent a single SRS procedure except three cases with fractionation. The single fraction SRS was was mostly performed with a marginal dose of 12.0 to 13.0 Gy to the 50% isodose lines. The fractionated SRS procedures were performed with a marginal dose of 25.6 Gy over 5 fractions or 20.0 Gy over 3 fractions to the 50% isodose lines.

**Table 2:** Treatment factors and outcomes.

	**VS (n=41) in NF2 patients**
Maximal extrameatal diameter (cm)	2.0 [1.1, 2.3]
Extrameatal extension (Koos grade)	
I	5 (12)
II	15 (37)
III	16 (39)
IV	5 (12)
Target volume (cc)	3.06 [1.00, 6.00]
Number of fractions	
Single	39 (93)
Fractionated	3 (7)
Marginal dose (Gy) per fraction	12.0 [12.0, 12.5]
Less than 12.0	5 (12)
12.0-13.0	35 (86)
More than 13.0	1 (2)
Tumor control	
At 12 months	24 (59)
At 24 months (n=36)	21 (58)
At 36 months (n=35)	26 (74)
At 60 months (n=27)	23 (85)
At 120 months (n=11)	9 (82)
Pseudoprogression (n=12)	
Peak volume increase (%)	29.2 [22.4, 36.6]
Time-to-peak volume (month)	17.3 [11.0, 35.4]
Hearing preservation (n=10)	
Serviceable	8 (80)
Non-serviceable	2 (20)
Other comorbidities	
Vestibulopathy	.
Facial nerve palsy	.
Trigeminal neuralgia	.
Symptomatic hydrocephalus (n=33)	1 (3)

Values are numbers (%) or a median [range]. Percentages are based on the total number of treated lesions (n=41), unless otherwise specified. VS, vestibular schwannoma; NF2, neurofibromatosis 2.

### Tumor growth patterns and tumor volume response to stereotactic radiosurgery

**Figure 2:**
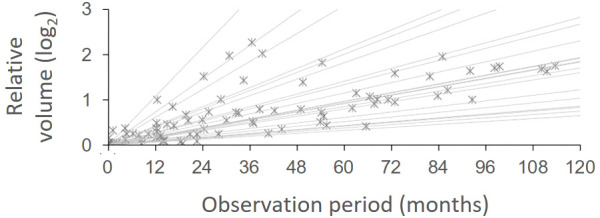
Growth patterns of unirradiated vestibular schwannomas. The relative volume change during the observation period was fitted using linear regression for each case [R^2^, 0.965 (IQR, 0.937-0.991)]. The volume of unirradiated lesions exponentially expanded with a doubling time of 62.2 (IQR, 30.6-92.3) months, which corresponds to a relative volume increase of 14.0% (IQR, 7.8-27.0%) per year.

To identify the natural course of the NF2-associated VS lesions in our present series, we conducted a longitudinal volumetric analysis of these tumors during the observation period before the initial irradiation. Among the individuals with bilateral disease, 8 cases that were later treated for the second lesion and 14 cases with untreated contralateral lesions were assessed with available pre-treatment MRI data from at least three different time points during the observation period of 83.4 (61.1-120.4) months. The lesions progressed with a relative volume increase of 14.0% (7.8-27.0) per year (**Figure 2**). For those 8 cases with both lesions being treated, SRS was performed for the second lesion at a median of 113.7 (40.8-185.2) months from the initial treatment. The decision to conduct this latter treatment was made after the lesion had grown to 3.57 (2.67-6.19) times its initial volume. No clinical factors were significantly associated with the growth rate.

**Figure 3:**
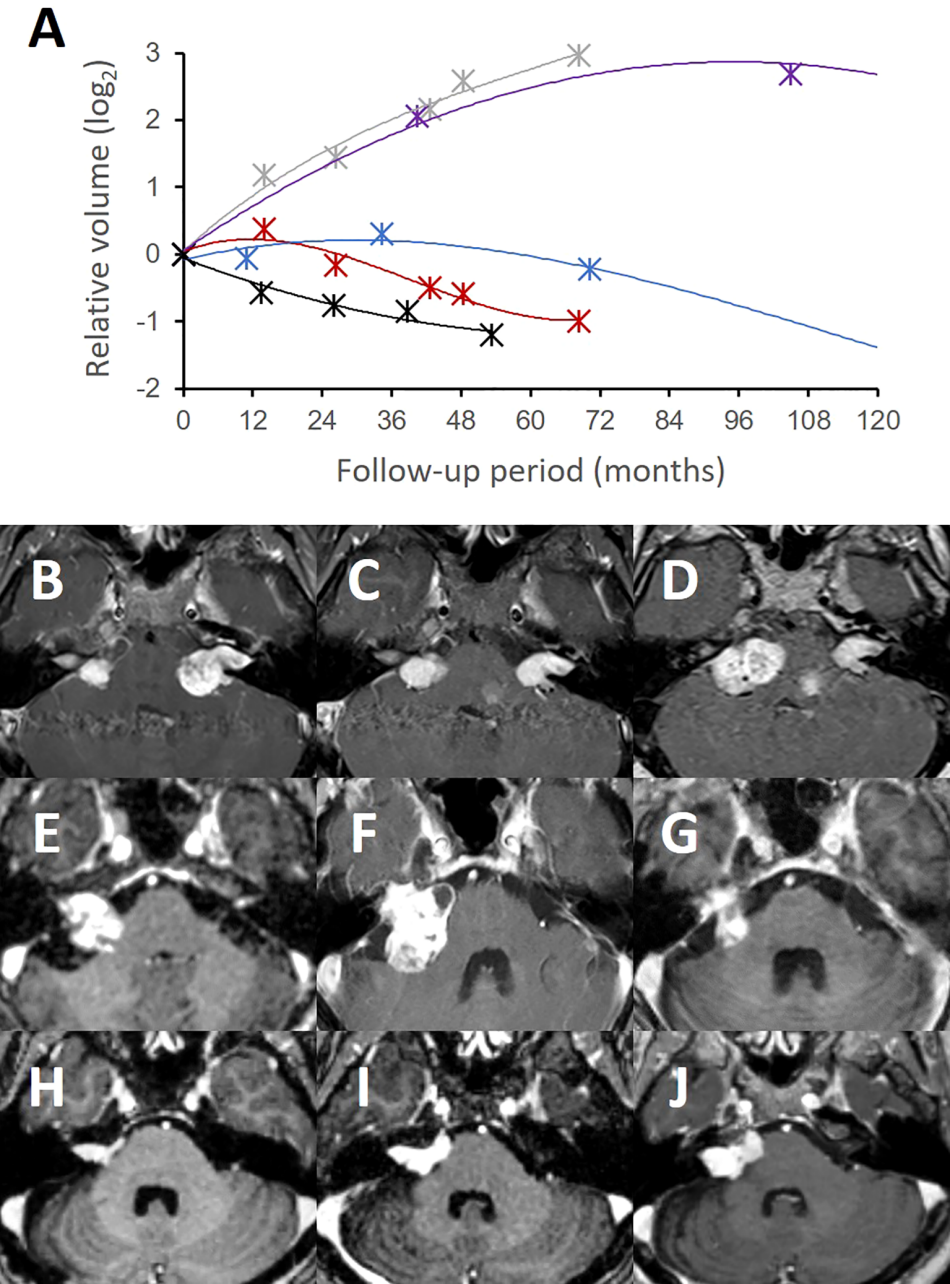
Tumor volume response patterns following stereotactic radiosurgery.The tumor volumes of the treated lesions showed distinct patterns in several treatment response groups **(A)**. Group I (black) refers to a typical volume response to SRS without significant pseudoprogression. Group II (red) volume responses had early pseudoprogression on the treated side compared to the contralateral untreated side (grey). Group III (blue) showed a slower response to SRS with delayed pseudoprogression. Group IV (purple) showed a failed tumor control with an exponential tumor growth pattern that was similar to untreated lesions (grey). A group I bilateral NF2-associated VS **(B–D)** showed favorable tumor control on the treated side (left). Notably, however, the contralateral untreated lesion (right) rapidly grew during the same follow-up period. A group III case **(E)** exhibited a slow treatment response, but successful tumor control over a long-term follow-up. This tumor had delayed pseudoprogression **(F)** up to three years post-treatment but eventually regressed without further treatment at 10 years **(G)**. A group IV case **(H)** was resistant to the SRS treatment and consistently grew over three years **(I)** and thereafter **(J)**.

We next investigated the tumor volume responses to the SRS procedure. We categorized each study patient into distinct treatment response groups in accordance with their individual tumor growth curves derived from longitudinal volumetric measurements (**Figure 3A**). Half (19 of 41) of these cases exhibited continuous volume shrinkage without pseudoprogression after the treatment (group I). Among the others who had significant volume expansion, 8 (20%) patients showed early pseudoprogression mostly within the first two years (group II; **Figures 3B–D**), whereas four (10%) cases showed late pseudoprogression with a peak volume at three or four years (group III; **Figures 3E–G**). Four (10%) cases where true tumor progression was suspected showed sequential tumor growth (group IV; **Figures 3H–J**) as found in the untreated lesions. Six cases were undetermined with respect to their response groups due to a follow-up period shorter than five years.

### Clinical course following stereotactic radiosurgery

The treated lesions were followed up for a median of 69.1 (45.0-104.8) months after SRS. Although four (10%) of our cases displayed failed tumor control at the time of the last imaging study, no cases required salvage surgery during the follow-up period (**Table 2**). The rate of tumor control varied across different time points from 56% at 12 months to 84% at 60 months. This rate was slightly lower in the earlier follow-up period within two years due to several cases presenting with pseudoprogression. Among the 12 cases that we identified as having pseudoprogression, the tumor volumes were found to have increased to 1.29 (1.22-1.37) times their initial volume. The time to peak volume was 17.3 (11.0-35.4) months. Nevertheless, the tumor volume had recovered to within the control limit in 34.5 (25.8-51.8) months in these cases.

**Figure 4:**
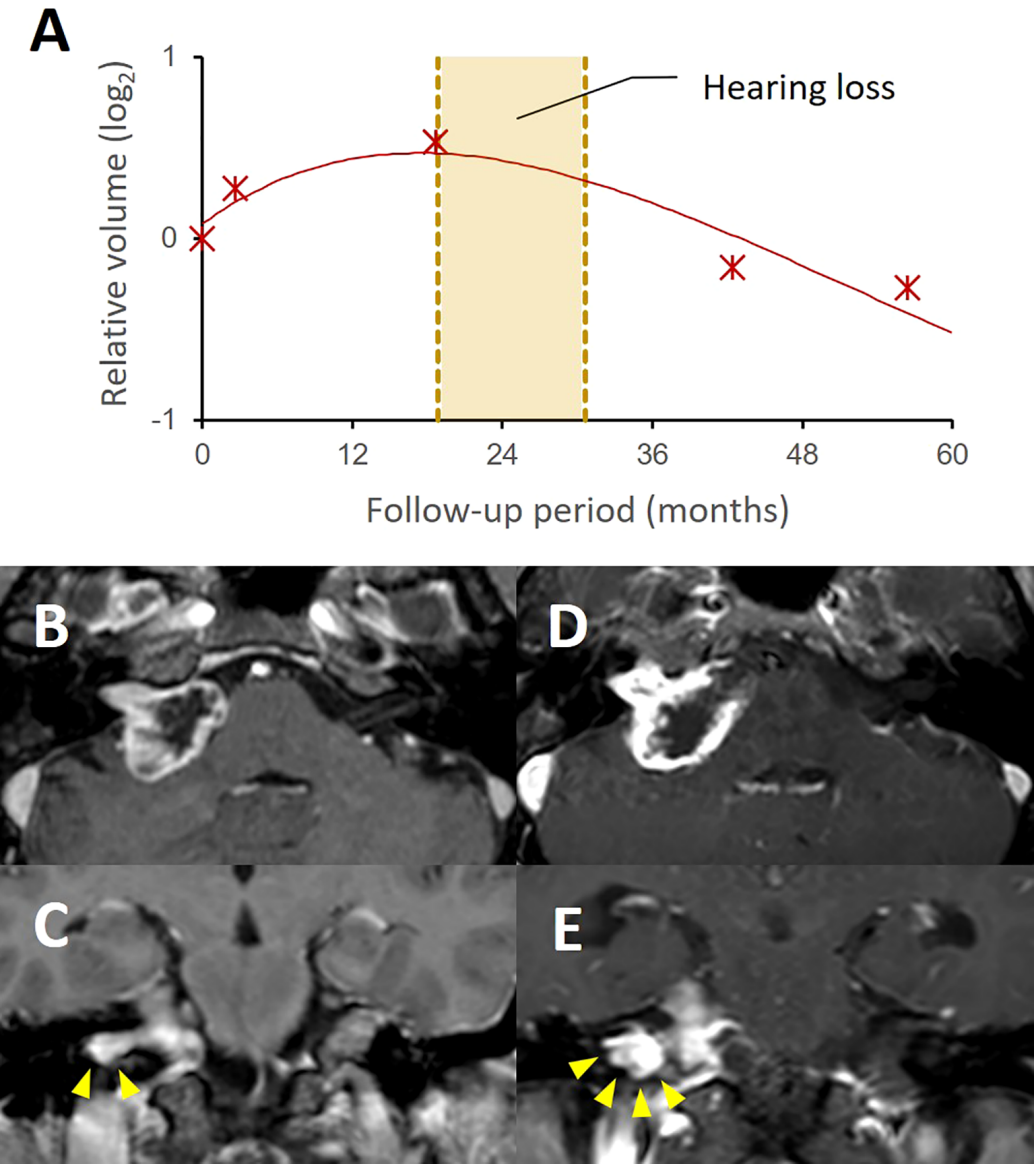
A case with progressive hearing loss during pseudoprogression after stereotactic radiosurgery. A group II case showed a typical tumor volume response pattern with transient volume increase up to 42.5% of initial volume after SRS **(A)**. The patient presented progressive hearing loss during pseudoprogression (yellow box). The follow-up MRI **(D, E)** showed a significant volume expansion, compared to the pretreatment MRI **(B, C)**. In this case, the increased extent of the intracanalicular part of the tumor was notable as shown in the coronal images (**E**, arrowheads), which possibly caused cochlear nerve compression in the internal auditory canal.

In the context of functional outcomes, hearing preservation was achieved in most (8 of 10) of our current study cases. One unilateral VS patient presented hearing loss on the treated side from Gardner-Robertson grade II to grade IV during early transient progression in 24 months (**Figure 4**). Another bilateral VS patient who had already lost hearing function on the contralateral side presented hearing impairment in 20 months, but the patient’s hearing recovered to Gardner-Robertson grade II after cochlear implantation. Our case series also included three patients with borderline function (Gardner-Robertson grade III), but none of them showed hearing improvement during the audiometric follow-up and two eventually lost their function (Gardner-Robertson grade IV/V) in 12 and 24 months, respectively. No cases presented with any of other cranial nerve dysfunction during follow-up. One subject presented with symptomatic communicating hydrocephalus.

## Discussion

VS is a common benign tumor and the basis of its treatment at present is focused on functional outcomes (1). Accordingly, optimal timing for the intervention and the treatment modality itself are important issues for practitioners. Previous studies have consistently reported on VS tumor growth and the risk of hearing loss in affected patients who undergo observation only without treatment. A prior nationwide cohort study revealed that untreated VS lesions usually grow within five years after their initial diagnosis, and 25-40% of them are still capable of growth after 10 years (13). Another large cohort study reported that 43% of NF2 patients start treatment for VS within five years of its diagnosis (3). The five-year mortality of NF2 patients has been reported to be up to 6% in previous articles (3, 14). The previous meta-analysis has also found that patients on an initial observation management plan have a higher risk of early hearing deterioration (15). Considering these circumstances, observation only may no longer be appropriate if there is any suspicion of interval tumor growth.

SRS is a possible good treatment option with an expectation of tumor control without facial palsy, in both non-NF2 (16) and NF2 (2) populations. There are however some concerns regarding the efficacy of this treatment, as some authors have suggested that the reported rates of tumor control have likely been overestimated due to a liberal definition and that SRS had no discernable volume reduction effects (17). Most previous studies have presented an excellent tumor control rate of more than 80% with SRS (6, 7, 12, 18, 19), but these numbers should be carefully assessed because they might overlook the clinical significance of pseudoprogression during the early follow-up period. In our present case series, 85% of the patients achieved mid- to long-term VS tumor control following SRS, which was comparable to the results described in previous reports (20). However, we also found in our present analyses that one-third of the VS lesions had expanded in volume to a considerably high level and that the tumor control rates at the early time points, i.e., within two years, were practically less than 60%. Nevertheless, in the NF2 population where VS tumors grow more rapidly than in sporadic cases, even an arrest of tumor growth could be advantageous. Currently, a combined treatment strategy of attempted subtotal resection followed by adjuvant SRS in large VS patients is widely accepted with satisfactory outcomes for facial and cochlear functions (21). These concepts for optimal tumor control can be also utilized in NF2 populations depending on the individual patient’s condition.

In the context of functional outcomes, the role of SRS has been limited due to the controversial results regarding hearing preservation (22). Most NF2 patients eventually present deafness in their lifetime, and most treatment modalities are rarely effective to revert the natural course of hearing dysfunction. It has been reported that NF2-associated VS is associated with a lower probability of hearing preservation following SRS treatment than sporadic VS (12, 18, 19). We also experienced a couple of cases in our current series in which hearing function was not maintained but in fact rapidly worsened during the early follow-up period. In our study, several cases showed considerably high volume expansion following the SRS treatment. It has been reported that a recent rapid progression with a greater growth rate is timely difficult to reverse using SRS (23). In addition, there might be other oncological or neuromodulatory mechanisms from the SRS procedure that possibly cause hearing deterioration. Hence, SRS should be carefully considered if hearing preservation is important for the patient. Further investigations that involve longitudinal volumetric analysis and physiologic characterization of VS among the NF2 population might provide better insights into the selection of candidates for SRS.

## Conclusions

Progressive NF2-associated VS can be well controlled by SRS, which restricts the growth of these tumors compared to patients who are not treated. Notably, however, the short-term treatment effects of SRS in these cases are not highly advantageous to preserving hearing functions. NF2 candidates should therefore be more carefully selected for this intervention than sporadic VS cases.

## Data availability statement

The original contributions presented in the study are included in the article/supplementary material. Further inquiries can be directed to the corresponding author.

## Ethics statement

The studies involving human participants were reviewed and approved by the institutional review board in Asan Medical Center. Written informed consent for participation was not required for this study in accordance with the national legislation and the institutional requirements.

## Author contributions

JK and Y-HK contributed to the conception and design of the study. JK performed the data analysis and wrote the first draft of the manuscript. All authors contributed to manuscript revision, read, and approved the submitted version.

## References

[1] Goldbrunner R Weller M Regis J Lund-Johansen M Stavrinou P Reuss D. EANO guideline on the diagnosis and treatment of vestibular schwannoma. Neuro Oncol (2020) 22(1):31–45. doi: 10.1093/neuonc/noz153 31504802 PMC6954440

[2] Chung LK Nguyen TP Sheppard JP Lagman C Tenn S Lee P. A systematic review of radiosurgery versus surgery for neurofibromatosis type 2 vestibular schwannomas. World Neurosurg (2018) 109:47–58. doi: 10.1016/j.wneu.2017.08.159 28882713

[3] Forde C King AT Rutherford SA Hammerbeck-Ward C Lloyd SK Freeman SR. Disease course of neurofibromatosis type 2: A 30-year follow-up study of 353 patients seen at a single institution. Neuro Oncol (2021) 23(7):1113–24. doi: 10.1093/neuonc/noaa284 PMC8248850 33336705

[4] Goutagny S Bah AB Parfait B Sterkers O Kalamarides M. Neurofibromatosis type 2 in the elderly population: clinical and molecular features. Am J Med Genet A (2013) 161A(4):667–70. doi: 10.1002/ajmg.a.35851 23322716

[5] Kim BS Seol HJ Lee JI Shin HJ Park K Kong DS. Clinical outcome of neurofibromatosis type 2-related vestibular schwannoma: treatment strategies and challenges. Neurosurg Rev (2016) 39(4):643–53. doi: 10.1007/s10143-016-0728-5 27142681

[6] Kruyt IJ Verheul JB Hanssens PEJ Kunst HPM. Gamma knife radiosurgery for treatment of growing vestibular schwannomas in patients with neurofibromatosis type 2: A matched cohort study with sporadic vestibular schwannomas. J Neurosurg (2018) 128(1):49–59. doi: 10.3171/2016.9.JNS161463 28128697

[7] Shinya Y Hasegawa H Shin M Sugiyama T Kawashima M Takahashi W. Long-term outcomes of stereotactic radiosurgery for vestibular schwannoma associated with neurofibromatosis type 2 in comparison to sporadic schwannoma. Cancers (Basel) (2019) 11(10). doi: 10.3390/cancers11101498 PMC6827030 31591325

[8] Evans DG Trueman L Wallace A Collins S Strachan T. Genotype/phenotype correlations in type 2 neurofibromatosis (NF2): Evidence for more severe disease associated with truncating mutations. J Med Genet (1998) 35(6):450–5. doi: 10.1136/jmg.35.6.450 PMC1051337 9643284

[9] Fouard O Daisne JF Wanet M Regnier M Gustin T. Long-term volumetric analysis of vestibular schwannomas following stereotactic radiotherapy: Practical implications for follow-up. Clin Transl Radiat Oncol (2022) 33:1–6. doi: 10.1016/j.ctro.2021.12.003 34977365 PMC8688865

[10] Meijer OW Weijmans EJ Knol DL Slotman BJ Barkhof F Vandertop WP. Tumor-volume changes after radiosurgery for vestibular schwannoma: Implications for follow-up MR imaging protocol. AJNR Am J Neuroradiol (2008) 29(5):906–10. doi: 10.3174/ajnr.A0969 PMC8128568 18296549

[11] Ragge NK. Clinical and genetic patterns of neurofibromatosis 1 and 2. Br J Ophthalmol (1993) 77(10):662–72. doi: 10.1136/bjo.77.10.662 PMC504612 8218038

[12] Sun S Liu A. Long-term follow-up studies of gamma knife surgery for patients with neurofibromatosis type 2. J Neurosurg (2014) 121 Suppl:143–9. doi: 10.3171/2014.8.GKS141503 25434947

[13] Reznitsky M Petersen M West N Stangerup SE Caye-Thomasen P. The natural history of vestibular schwannoma growth-prospective 40-year data from an unselected national cohort. Neuro Oncol (2021) 23(5):827–36. doi: 10.1093/neuonc/noaa230 PMC8099466 33068429

[14] Goshtasbi K Abouzari M Yasaka TM Soltanzadeh-Zarandi S Sarna B Lin HW. Treatment analysis and overall survival outcomes of patients with bilateral vestibular schwannoma. Otol Neurotol (2021) 42(4):592–7. doi: 10.1097/MAO.0000000000002984 PMC8080845 33351555

[15] Leon J Lehrer EJ Peterson J Vallow L Ruiz-Garcia H Hadley A. Observation or stereotactic radiosurgery for newly diagnosed vestibular schwannomas: A systematic review and meta-analysis. J Radiosurg SBRT (2019) 6(2):91–100. doi: 10.1016/j.wneu.2021.11.083 31641546 PMC6774488

[16] Savardekar AR Terrell D Lele SJ Diaz R Keesari PR Trosclair K. Primary treatment of small to medium (<3 cm) sporadic vestibular schwannomas: A systematic review and meta-analysis on hearing preservation and tumor control rates for microsurgery versus radiosurgery. World Neurosurg (2022) 160:102–13.e112. doi: 10.1016/j.wneu.2021.11.083 34838768

[17] Battaglia A Mastrodimos B Cueva R. Comparison of growth patterns of acoustic neuromas with and without radiosurgery. Otol Neurotol (2006) 27(5):705–12. doi: 10.1097/01.mao.0000226302.59198.87 16868519

[18] Spatola G Carron R Delsanti C Thomassin JM Roche PH Regis J. Long-term results of gamma-knife stereotactic radiosurgery for vestibular schwannomas in patients with type 2 neurofibromatosis. Neurochirurgie (2018) 64(5):355–63. doi: 10.1016/j.neuchi.2016.03.005 27527622

[19] Mallory GW Pollock BE Foote RL Carlson ML Driscoll CL Link MJ. Stereotactic radiosurgery for neurofibromatosis 2-associated vestibular schwannomas: Toward dose optimization for tumor control and functional outcomes. Neurosurgery (2014) 74(3):292–300. doi: 10.1227/NEU.0000000000000264 24335819

[20] Tosi U Maayan O An A Lavieri MET Guadix SW DeRosa AP. Stereotactic radiosurgery for vestibular schwannomas in neurofibromatosis type 2 patients: A systematic review and meta-analysis. J Neurooncol (2022) 156(2):431–41. doi: 10.1007/s11060-021-03910-8 35040021

[21] Starnoni D Giammattei L Cossu G Link MJ Roche PH Chacko AG. Surgical management for large vestibular schwannomas: a systematic review, meta-analysis, and consensus statement on behalf of the EANS skull base section. Acta Neurochir (Wien) (2020) 162(11):2595–617. doi: 10.1007/s00701-020-04491-7 PMC7550309 32728903

[22] Watanabe S Yamamoto M Kawabe T Koiso T Yamamoto T Matsumura A. Stereotactic radiosurgery for vestibular schwannomas: Average 10-year follow-up results focusing on long-term hearing preservation. J Neurosurg (2016) 125(Suppl 1):64–72. doi: 10.3171/2016.7.GKS161494 27903183

[23] Killeen DE Tolisano AM Isaacson B Kutz JW Barnett S Wardak Z. Vestibular schwannoma tumor size and growth rate predict response with gamma knife stereotactic radiosurgery. J Neurol Surg B Skull Base (2022) 83(1):11–8. doi: 10.1055/s-0040-1716677 PMC8824627 35155064

