# Corrigendum: Vestibular schwannoma associated with neurofibromatosis type 2: Clinical course following stereotactic radiosurgery

**DOI:** 10.3389/fonc.2022.1100854

**Published:** 2022-12-08

**Authors:** Junhyung Kim, Yukyeng Byeon, Sang Woo Song, Young Hyun Cho, Chang-Ki Hong, Seok Ho Hong, Jeong Hoon Kim, Do Heui Lee, Ji Eun Park, Ho Sung Kim, Young-Hoon Kim

**Affiliations:** ^1^ Department of Neurological Surgery, Asan Medical Center, University of Ulsan College of Medicine, Seoul, South Korea; ^2^ Department of Radiology, Asan Medical Center, University of Ulsan College of Medicine, Seoul, South Korea

**Keywords:** neurofibromatosis type 2 (NF2), vestibular schwannoma (acoustic neuroma), stereotactic radiosurgery (SRS), tumor control, hearing preservation

In the published article, there was a misprint in affiliations. Instead of “Department of Neurosurgery, Asan Medical Center, Seoul, South Korea” it should be “Department of Neurological Surgery, Asan Medical Center, University of Ulsan College of Medicine, Seoul, South Korea”. Additionally, instead of “Department of Radiology, Asan Medical Center, Seoul, South Korea” it should be “Department of Radiology, Asan Medical Center, University of Ulsan College of Medicine, Seoul, South Korea”.

The authors apologize for this error and state that this does not change the scientific conclusions of the article in any way. The original article has been updated.

